# Dynamics of Viral and Host 3D Genome Structure upon Infection

**DOI:** 10.4014/jmb.2208.08020

**Published:** 2022-09-30

**Authors:** Meyer J. Friedman, Haram Lee, Young-Chan Kwon, Soohwan Oh

**Affiliations:** 1 Department and School of Medicine, University of California, San Diego, La Jolla, CA 92093, USA; 2 College of Pharmacy, Korea University, Sejong 30019, Republic of Korea; 3 Center for Convergent Research of Emerging Virus Infections, Korean Research Institute of Chemical Technology, Daejeon 34114, Republic of Korea

**Keywords:** Chromatin structure, EBV, KSHV, HPV, HIV, influenza virus, SARS-CoV-2

## Abstract

Eukaryotic chromatin is highly organized in the 3D nuclear space and dynamically regulated in response to environmental stimuli. This genomic organization is arranged in a hierarchical fashion to support various cellular functions, including transcriptional regulation of gene expression. Like other host cellular mechanisms, viral pathogens utilize and modulate host chromatin architecture and its regulatory machinery to control features of their life cycle, such as lytic versus latent status. Combined with previous research focusing on individual loci, recent global genomic studies employing conformational assays coupled with high-throughput sequencing technology have informed models for host and, in some cases, viral 3D chromosomal structure re-organization during infection and the contribution of these alterations to virus-mediated diseases. Here, we review recent discoveries and progress in host and viral chromatin structural dynamics during infection, focusing on a subset of DNA (human herpesviruses and HPV) as well as RNA (HIV, influenza virus and SARS-CoV-2) viruses. An understanding of how host and viral genomic structure affect gene expression in both contexts and ultimately viral pathogenesis can facilitate the development of novel therapeutic strategies.

## Introduction

Distal cis-regulatory enhancers control appropriate transcription of genes in eukaryotes through their interaction with proximal cis-regulatory promoters. These DNA regulatory elements, which exhibit distinctive epigenetic features, can be separated by tens to hundreds of kilobases (kb) and contain binding sequences for specific transcription factors (TFs) that drive gene expression in part by recruiting co-factors endowed with various enzymatic activities responsible for altering the epigenetic landscape. Advances in the gene regulation field have revealed that 3D chromatin (re-)organization plays an important role in bringing enhancers in close proximity to their target promoters [1]. The structure of the 3D genome largely relies on architectural proteins such as CTCF, cohesin, and YY1 [2]. These architectural proteins contribute not only to topologically associated domains (TADs), which are demarcated by boundaries, but also to smaller chromatin loops within TADs, such as enhancer-promoter (E-P) interactions. In addition to the characteristic epigenetic properties of enhancers and promoters, CTCF/cohesin effects on 3D chromatin organization are a key determinant of E-P interaction specificity and thus spatiotemporal regulation of gene expression [3, 4]. Intriguingly, depletion of host architectural proteins, including CTCF or cohesin, also affects reactivation and replication of many viruses (Table 1). These results imply that the viral life cycle can be regulated by chromosome architecture and further suggest viral mechanisms involving genome organization during infection.

Multiple lines of evidence indicate that the infection state (*i.e.*, lytic vs. latent) significantly impacts chromosome structure as well as E-P interactions of the host and can involve distinct viral-host genomic interplay. Recent advances in genome-wide chromatin biology have revealed a crucial role for chromatin architecture and epigenetic signatures in the regulation of viral infection. It is now apparent that the genomic conformations of both the host and the virus collectively influence the respective gene expression profiles. DNA viruses, some of which can integrate whereas others are maintained as multicopy minichromosomes (or episomes), have several mechanisms that control genome organization of the host and themselves. RNA viruses, a subset of which can also integrate, utilize additional mechanisms for modulating host genome organization involving viral gene products or hijacked host regulatory machinery (Table 2). This review seeks to provide an informative summary of our current understanding of host and viral genomic structural dynamics and its relationship with gene expression in infected cells.

## Technologies for Investigating Chromosome Architecture

**Fig. 1:**
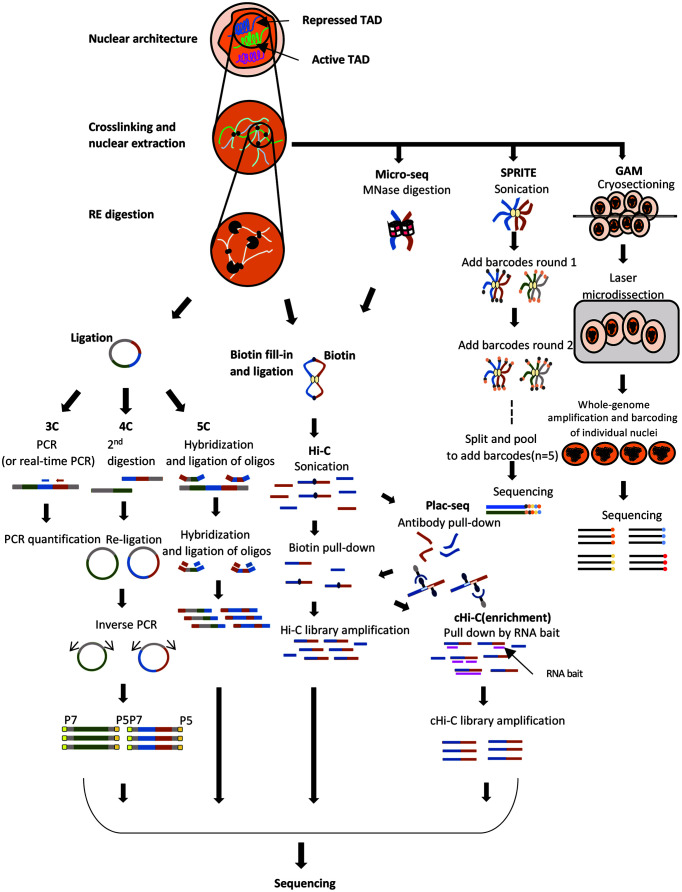
Schematic representation of assays for studying chromosome architecture. For 3C-based methods, nuclei are first treated with appropriate fixatives (*i.e.*, formaldehyde, DSG, etc.). In GAM, cryosections are cut from paraformaldehydefixed and sucrose-embedded samples. In 3C, 4C and 5C, fixed nuclei are treated with restriction enzymes, ligated, and the ligation frequency is measured by PCR or NGS. For Hi-C, Plac-seq, and cHi-C, chromosomal DNA is digested by restriction enzymes while micro-C uses MNase for finer resolution. The digested DNA ends are repaired with biotin-labeled nucleotides followed by blunt-end ligation. The ligated biotin-labeled contacts are sheared and purified with streptavidin beads prior to NGS sequencing. In Plac-seq and cHi-C, antibody pull-down or RNA oligo-mediated DNA pull-down is performed for target enrichment, respectively. In the SPRITE method, crosslinked chromatin is fragmented by sonication, each interacting complex is uniquely tagged by multiple rounds of split-pool barcoding, and the final material is sequenced. In the GAM method, the DNA contents from cryosections are extracted, fragmented, and sequenced. Appropriate computational analysis of the sequencing data from each approach is necessary to detect physical interactions between genomic loci.

The advent of chromosome conformation capture (3C)-based techniques coupled with next-generation sequencing (NGS) technology has dramatically elevated our appreciation of chromosomal organization (Fig. 1). A series of 3C-derivative sequencing methods involving crosslinking, enzymatic digestion of chromosomes, and proximity ligation have enabled high-throughput and genome-wide detection of contact frequency between genomic loci, including Circular Chromosome Conformation Capture (4C) [5] (one to all), Chromosome Conformation Capture Carbon Copy (5C) [6] (many to many), capture-HiC [7], Proximity Ligation-Assisted ChIP-Seq (PLAC-seq) [8], Chromatin Interaction Analysis by Paired-End Tag Sequencing (ChIA-PET) [9] (many to all), Hi-C [10] and Micro-C [11] (all to all). All of these 3C-based techniques, except for Micro-C, use restriction enzymes to achieve the desired resolution. In principle, a more frequent cutter will yield higher resolution. However, even with a 4-cutter enzyme, which recognizes a tetranucleotide sequence, information on finer contacts between genomic loci, such as E-P, is very rare. In Micro-C, improved resolution to the level of individual nucleosomes (147 bp) is achieved by employing micrococcal nuclease (MNase) in the digestion step. Accordingly, Micro-C structural information shows overall consistency with that of Hi-C but with increased power for detecting short-range associations, including E-P interactions. Several studies have interrogated host and, when applicable, viral chromosomal architecture upon infection with 3C-based techniques, but none have reported data with nucleosomal resolution.

Ligation-independent methods that retain a crosslinking step also have been introduced to assess 3D genomic organization, including split-pool recognition of interactions by tag extension (SPRITE) [12] and genome architecture mapping (GAM) [13]. SPRITE relies on the sequencing of barcoded DNA following multiple rounds of splitting and pooling, such that each interacting chromatin complex is expected to have a unique barcode. The interaction map can be determined from the extracted DNA segments with the same barcode. In the GAM technique, interaction information is derived from micro-dissected slices of nuclei in fixed cells. Because DNA loci in close proximity have a higher probability of being in the same slice, the frequency of the genomic regions in a given slice is calculated as the interaction map. Currently, there are no published studies using SPRITE or GAM to interrogate viral and host genomic organization.

In some cases, the viral components of NGS datasets have not been analyzed yet. For example, lymphoblastoid cell lines (LCLs), which are widely-used, immortalized B cells, are established by infection with the human herpesvirus (HHV) EBV. Because of the convenience of immortalization, the HapMap project [14] used LCLs to amass genotype and gene expression data. In addition, extensively characterized tier 1 ENCODE cell lines include an LCL designated as GM12878 [15]. In LCLs, ~1% of mappable sequencing reads from RNA-seq, ChIP-seq, and other NGS datasets can be aligned to the EBV genome [16]. Thus, a substantial amount of NGS data pertaining to epigenetic profiles and chromosome organization have been collected for the EBV genome through HapMap, ENCODE, and subsequent studies [17, 18]. By virtue of these publicly available datasets, EBV chromatin biology has been relatively well studied compared to other viruses.

## Herpesviruses

Herpesviruses are double-stranded DNA viruses that have the potential to establish lifelong infection and cause various diseases. The eight HHVs (HHV1-8) feature relatively large DNA genomes of more than 100kb that encode 100-200 genes.

After infection, the HHV genomes circularize and undergo chromatinizing events, such as histone binding and DNA methylation. The chromatinized viral genome acts as a minichromosome, which is referred to as an episome, and recruits host chromosome regulatory machinery [19, 20]. Because HHV episomes behave like host chromosomes, the role of 3D chromatin structure on viral events and pathogenesis has been extensively studied. In EBV (HHV4) and KSHV (HHV8), the chromosome architectural proteins CTCF and cohesin co-occupy several genomic loci. CTCF/cohesin co-bound sites are most enriched at terminal repeats and in the vicinity of key latent transcripts [16, 21]. Since CTCF/cohesin binding to chromosomes facilitates the formation of chromatin loops, including TADs and E-P interactions, a number of studies have attempted to uncover the role of chromatin conformation in transcriptional control of HHV infection-associated gene expression (Tables 1, 2), as highlighted below.

### EBV Episomal Structure

EBV-infected B cells are transformed to LCLs via EBV nuclear antigens (EBNAs). Of the six EBNAs, EBNA2 is specifically important for the expression of essential host and viral genes required for the establishment and proliferation of LCLs and hence EBV persistence [22]. Chromatin conformation has a significant role in the regulation of EBNAs. A loop between the shared EBNA gene promoter Cp and the viral origin of replication (OriP) has an enhancer-blocking effect that silences EBNA2 expression upon transition to restricted latency, which is characterized by increasingly limited latent viral transcription [23, 24]. Formation of this loop depends on CTCF occupancy of a binding site in the EBV genome [24], deletion of which results in upregulation of EBNA2 expression [23]. Conversely, CTCF-dependent transcriptional activation also has been reported in the EBV genome. Disruption of the CTCF binding site in the latent Q promoter (Qp) attenuates associated transcription [25]. In addition, mutagenesis of the CTCF binding site positioned within the overlapping EBV *LMP1* and *LMP2A* genes reduces their expression as part of the observed deregulation of the perturbed latent viral locus [26].

Modulation of viral gene expression through episome structural reconfiguration can affect host chromosome conformation and transcriptional regulation. Latent EBNA proteins serve as transcription factors not only for the viral genome but also for host genes, as exemplified by EBNA2 induction of the *MYC* proto-oncogene to promote B cell proliferation [27, 28]. EBNA2 binds to distal enhancers located hundreds of kb from the transcriptional start site (TSS) of *MYC* [29]. Notably, inactivation of EBNA2 reduces MYC expression by weakening chromatin looping between EBNA2-binding enhancers and the *MYC* TSS [30]. Furthermore, MYC upregulation increases its recruitment to cognate E-box motifs located proximal to the origin of lytic replication (*oriLyt*) in the EBV genome. MYC occupancy prevents *oriLyt* interaction with the promoter of the immediate early lytic factor BZLF1 to preserve viral latency [31].

### KSHV Episomal Structure

Studies of KSHV show strong CTCF/cohesin binding at an intergenic region between the latent gene *LANA* and the lytic gene *K14* [32]. Disruption of this CTCF/cohesin binding induces the expression of a set of lytic genes, including *K14*, *ORF74*, *ORF57*, and *ORF6* [33, 34]. CTCF/cohesin binding is also detected at the promoter of the reactivation gene *ORF50/RTA*, which is upregulated upon depletion of cohesin [21]. These results raise the possibility that CTCF/cohesin-mediated chromosome architecture can restrict KSHV lytic gene expression and replication. Like EBV, KSHV also increases proto-oncogene MYC expression in an enhancer-dependent manner; however, the viral activator of *MYC* enhancers in the host genome remains elusive [35].

### Episomal Structure of Other Herpesviruses

In HSV-1(HHV1) and CMV(HHV5), analogous to other HHVs, CTCF interacts with the viral genome, and it can serve as an insulator to regulate gene expression in the latent and reactivation states [36-39]. In addition, CTCF is recruited to the genome of lytic HSV-1 at multiple binding sites whereupon it promotes lytic transcription by facilitating RNA Pol II elongation and by preventing the deposition of silencing chromatin marks [40]. While cohesin has been reported to support HSV1 lytic transcription in a similar fashion to CTCF [41], any contribution of CTCF/cohesin-mediated episome structural effects has not been rigorously evaluated in the lytic context.

### NGS-Based Studies of Herpesvirus and Host Chromatin Structure

Recently, a number of studies have sought to interrogate HHV episomal structure by application of unbiased, genome-wide chromosomal architecture methods, such as Hi-C.

**Insights into herpesvirus episomal structure**. Re-analysis of EBV-aligned reads in Hi-C data from the LCL cell line GM12878 [17], which features the most transcriptionally active latency program, latency III, confirmed clustering of the major EBNA gene promoter Cp, the latent promoter Qp, and OriP [42]. Further comparison of GM12878 with the largely transcriptionally silent LCL Mutu, representing latency I, revealed significantly different viral episome structures, including substantially more intragenomic contacts in type III than type I latency. Interestingly, EBV episomal structure relies on poly(ADP-ribose) polymerase (PARP) enzymatic activity, which stabilizes CTCF occupancy but may antagonize cohesin binding. Inhibition of PARP1, a transcriptional co-factor and the most active PARP family member, leads to fewer loops within the EBV episome and, curiously, decreased CTCF but increased cohesin at their established co-binding sites in the viral genome [42].

Capture-HiC of the KSHV episome revealed a 3D viral genomic map with a number of dynamic, long-range interactions within the viral chromosome [43]. This study revealed a tendency for gene clustering within similar functional genomic domains, such as the distinct latency and early-lytic gene clusters. Upon lytic protein K-Rta-induced reactivation, genomic interaction of regions encoding early- and immediate-early lytic genes is augmented. A subsequent capture-HiC study with finer resolution (500 bp) reported the KSHV TAD structure, which is restricted by CTCF/cohesin binding [44]. During reactivation, K-Rta is recruited to TAD boundaries, leading to the formation of a larger regulatory unit with a shift from repressive B compartments to active A compartments. However, in these studies, terminal repeats (TRs, >30 repeats of 0.8kb genomic sequence for a total length of ~30 kb), which are likely key regulatory and architectural genomic features, were excluded during analysis due to the technical challenge of mapping repetitive sequences. Therefore, future studies will need to address the impact of TRs on episomal conformation.

**Assessment of host and herpesvirus chromosome interaction.** Extensive chromosomal interaction between the host and HHV genomes also has been documented recently. In situ Hi-C of EBV-infected Burkitt lymphoma (BL) cell lines revealed inter-chromosomal interactions between host chromosomes and the viral episome during latent infection [45], including preferential association of the EBV genome with inactive and low-gene density regions of the host genome. In addition, 4C-seq using EBV sequence as the viewpoint identified genome-wide association of EBV episomes and host chromosomes in BL [46]. In latently infected BL cells, the EBV episome favors interaction with host genomic sites bound by EBNA1, which tend to be transcriptionally silenced. In infected cells of different origins, the latent EBV episome also makes frequent contacts with lamina-associated domains (LADs) in the host genome, which generally constitute heterochromatic environments with limited transcriptional activity [45, 47]. Thus, the nuclear lamina may be a critical topological and epigenetic regulator of the EBV episome [48]. Interestingly, EBV-host chromosomal interactions are altered during viral reactivation from latency, as the viral episomes reposition from transcriptionally repressive heterochromatin toward active euchromatin [45].

Genome-wide analysis of 3D chromatin topologies in EBV-infected gastric cancer (GC) cells indicated a transition from heterochromatin to euchromatin at EBV-interacting host genomic loci [47]. EBV episomes associate with repressed H3K9me3-marked host enhancers, which are reprogrammed to engage and activate nearby GC-related proto-oncogenes. In other studies, re-analyzed Hi-C [17] and 4C-seq datasets from LCLs showed evidence of EBV episomes interacting with typical and super enhancers decorated with active epigenetic marks in the host genome [49]. Inconsistencies regarding the reported localization as well as functional significance of host-viral chromosomal interactions in BL, GC, and LCLs might be attributable, at least in part, to cell type- and/or latency type-specific effects and regulatory strategies [46].

Similar to EBV, in situ Hi-C analysis suggested that latent KSHV episomes preferentially associate with heterochromatic, low-gene density regions of the host genome and tend to move toward active euchromatin upon reactivation [45]. In addition, capture-HiC performed in primary effusion lymphoma (PEL) cells indicated that host interaction sites for KSHV episomes are enriched near centromeres [50]. While the significance of this localization remains unclear, overlay of ChIP-seq results for identified cellular interactors of the KSHV latency protein LANA, which has a well-established role in episome maintenance in infected, replicating cells that it achieves by binding KSHV TRs in addition to host chromatin factors [51], suggested an intriguing regulatory strategy for viral latency. The data support a model in which latent KSHV episomes colocalize with LANA and cellular CHD4/ADNP-containing ChAHP complexes on host chromosomes through interactions involving KSHV TRs [50]. This interplay could promote viral latency by allowing the strong repressor protein CHD4 to suppress KSHV lytic genes. Furthermore, a viral lncRNA that is robustly upregulated during KSHV reactivation, known as PAN RNA, may titrate CHD4 from KSHV episomes to de-repress viral lytic genes [50].

### Herpesvirus Summary

**Fig. 2:**
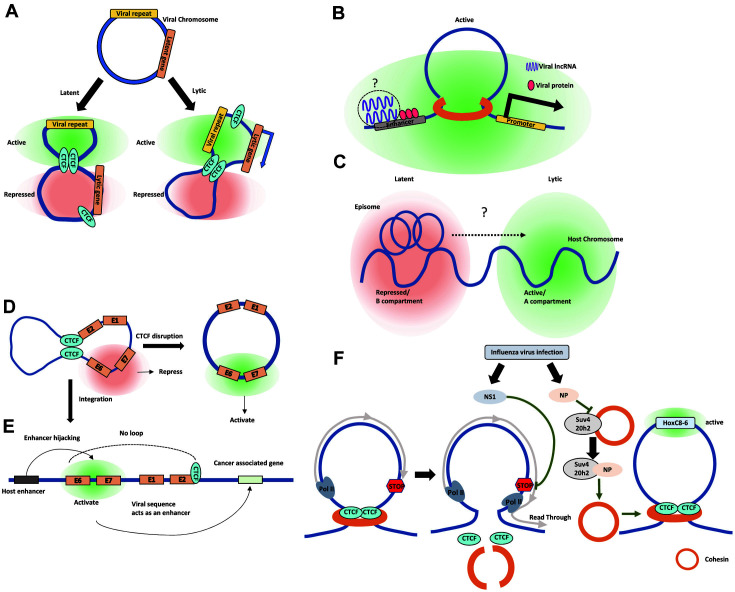
Models of viral and host regulation related to chromatin conformation. (**A**) In herpesviruses, host architectural proteins (*i.e.*, CTCF and cohesin) bind the viral episome and influence viral gene expression depending on the state of the virus (*i.e.*, latent vs. lytic) via conformational changes to the episome. (**B**) Viral gene products can modulate host gene expression by targeting host enhancers or promoters (*i.e.*, EBNAs and LANA). (**C**) The herpesvirus episome is associated with a repressed compartment in the host genome during latency. Upon reactivation, the interaction between the episome and euchromatin may increase. This viral-host chromosomal interplay could modulate both viral and host gene expression. (**D**) CTCF binding at the E2 promoter acts as a suppressor of viral oncogene E6/E7 by modulating HPV chromatin architecture. Disruption of CTCF binding causes transcriptional activation of E6/E7 in the HPV episome. (**E**) Viral integration into the host genome weakens looping between the E2 and E6/E7 loci, which also results in activation of the latter. The integrated viral sequence can hijack host enhancers for transcription of viral gene products or can act as an enhancer itself to stimulate neighboring cancer-associated genes. (**F**) Upon IAV infection, the viral protein NS1 inhibits the termination of transcription in a subset of highly transcribed genes. This inhibition leads to RNA Pol II-dependent dissociation of CTCF from its binding sites and disruption of chromatin loops (left). IAV protein NP competitively binds the host protein Suv4-20h2 to release Suv4-20h2- bound cohesin. Liberated cohesin is then recruited to the *HoxC8-HoxC6* locus where it induces gene expression by forming an active chromatin loop (right).

Studies of HHVs have revealed multiple mechanisms by which chromatin organization allows for maintenance and regulatory control of viral genomes as extrachromosomal DNA, or episomes, that extend beyond viral replication during interphase. Furthermore, host chromosome structural alterations, and their functional effects, are also an important feature of HHV-infected cells (Table 2). Possible molecular mechanisms involving chromosome conformation consequent to HHV infection include: 1) use of host chromosome architectural proteins by viruses to achieve changes in viral gene expression by modulating 3D episomal structure (Fig. 2A); 2) association of viral gene products with regulatory elements of host target genes to induce epigenetic/chromatin organization alterations that impact gene expression (Fig. 2B); and 3) interaction of virus and host chromosomes, the pattern of which can vary during the viral life cycle and selectively affects viral and host gene expression (Fig. 2C).

## Human Papillomavirus

Human papillomaviruses (HPVs) are a group of small, non-enveloped dsDNA viruses that are responsible for 99% of cervical cancer (CC) cases [52]. HPV genomes can be maintained in the nucleus either as a chromatinized episome or by integration into the host genome. HPV infection modulates host gene expression by various mechanisms, including direct effects on host chromosomal architecture and through the actions of viral oncoproteins E6 and E7 (E6/E7) [53, 54]. The HPV protein E2 acts as a negative regulator of E6/E7, and its disruption leads to overexpression of E6/E7 that promotes oncogenesis [55].

### HPV Episomal Structure

HPV viral gene expression is also regulated by host chromosome architectural proteins. A cohesin subunit, SMC1, is constitutively activated by HPV infection and binds to the HPV genome in a CTCF-dependent manner to facilitate genome amplification [56, 57]. Notably, the HPV genome harbors a conserved CTCF binding site in the *E2* ORF that recruits CTCF to regulate viral gene expression [58]. Disruption of this CTCF binding site results in decreased *E2* expression as well as epigenetic activation of an HPV enhancer, which collectively upregulates *E6/E7* to enhance cell proliferation. During keratinocyte differentiation, loss of CTCF/YY1-dependent suppressive looping in the HPV18 episome results in activation of viral enhancer-mediated E6/E7 expression [58, 59] and modulation of viral transcript splicing [60].

### HPV Integration

HPV integration into the host genome dramatically impacts chromosomal architecture with pathological consequences. Viral integration, which is a feature of most HPV-induced CCs, results in induction of oncogenic *E6/E7* due to downregulation of E1 and E2 [61]. Uniquely, HPV integration can cause structural variants (SV) of host chromosomes that result in gene expression changes [62], which are tightly linked to tumorigenesis [63]. Moreover, as demonstrated in HeLa cells, an extensively studied CC model system, HPV integration is directly associated with a long-range 3D genome organization that favors cancer development [64].

Although HPV integration sites are dispersed throughout the host genome, they tend to be enriched in transcriptionally active regions [65, 66]. In addition, several integration hotspots, which are associated with cancer-related gene expression programs, have been reported [62, 67]. HPV integration breakpoints occur more frequently near super enhancers, which can be amplified at sites of recurrent integration. Some of these super enhancers control the expression of cell-identity genes and may be co-opted to upregulate integrated viral oncogenes [68]. Moreover, integration events can yield super enhancer-like elements composed of tandem, interspersed copies of a viral and a cellular enhancer that drive viral oncogene expression [69]. Comprehensive evaluation of viral integration sites using Nanopore long-read sequencing uncovered various configurations of tandemly integrated HPV sequences at high resolution [70]. Analysis of 16 HPV-positive CCs revealed four HPV integration types, some of which can induce genomic disruptions through chromosomal translocation in addition to known effects on genes located proximal to integration breakpoints. Interestingly, the long-read sequencing data demonstrated that HPV can excise host genomic DNA along with integrated viral DNA to generate a viral-host hybrid extrachromosomal circular DNA (eccDNA) species. Because eccDNA may play an important role in CC by regulating oncogene expression [71], follow-up studies are needed for further characterization of these genomic hybrids.

### HPV-Induced Host Genome Structural Variants

Host genome SVs induced by HPV integration have been explored in an unbiased, genome-wide manner using 3C-based approaches. Hi-C data suggested that TAD alterations can be associated with enhancer hijacking, leading to changes in gene expression [54]. HPV integration at a hotspot in the *CCDC106* locus splits the affected TAD into two smaller TADs and, due to the newly imposed TAD boundaries, redirects an enhancer from the tumor suppressor gene *PEG3* to *CCDC106*, causing downregulation of the former and upregulation of the latter, which encodes a pro-proliferative factor. Another Hi-C study interrogating CCs also demonstrated significant alteration in 3D genome architecture, with A/B compartments switching in 24% of the host genome as compared to normal tissues [72]. Consistent with previous findings, genes neighboring HPV integration breakpoints were differentially expressed and strongly associated with various cancer-related pathways [54, 72]. Finally, modulation of host chromosome 3D structure by viral integration may be partially attributable to CTCF recruitment to conserved binding sites in the viral genome, resulting in reorganization of chromatin interactions [73].

In summary, HPV episomal structure is largely dependent on CTCF occupancy, disruption of which can be sufficient to upregulate viral E6/E7 oncogenes (Fig. 2D). Notably, CTCF binding sites are often mutated upon viral integration, causing overexpression of E6/E7. The preference of viral integration sites for actively transcribing host genomic regions enables HPV to hijack host regulatory elements and machinery to drive viral gene expression. Alternatively, the integrated viral sequence can act as an enhancer or boundary element, leading to dysregulation of host gene expression via alteration of short- and long-range chromosomal interactions. The host genes that are impacted by the above viral mechanisms are often cancer-associated, and the expression changes of host and viral (onco)genes collectively contribute to the transformation of infected cells (Fig. 2E).

## HIV-1

Human immunodeficiency virus type 1 (HIV-1) is a retrovirus with a positive-sense, single-stranded RNA genome that can result in Acquired Immunodeficiency Syndrome (AIDS). Long-term maintenance of HIV-1 requires stable integration of the viral genome into a host chromosome via a viral integrase-dependent mechanism during its life cycle.

### HIV-1 Integration

HIV-1 preferentially integrates in active genes in euchromatin that selectively localizes in spatial proximity to the nuclear pore compartment [74, 75]. The integrated HIV-1 provirus can enter a latent state that is facilitated by the deposition of repressive chromatin marks (*i.e.*, H3K27 and H3K9 trimethylation) and concomitant transcriptional silencing. Alternatively, integrated HIV-1 proviruses can remain transcriptionally and translationally active, irrespective of replication capacity, which most have lost due to acquired deletions and other mutations. Differential transcription of individual proviruses has been assessed systematically with a sequencing-based approach, dubbed barcoded HIV ensembles (B-HIVE), which revealed position effects [76]. In these studies, proviral expression levels correlated best with the presence of a nearby host genomic enhancer (< 5kb). Furthermore, inhibiting the interaction between HIV-1 integrase and the chromatin-tethering factor LEDGF/p75, which associates with the H3K36me3 chromatin mark characteristic of actively transcribed genes, attenuated proviral expression and increased the amount of silent provirus, which tended to integrate further away from H3K36me3-marked sites [77]. These results indicate HIV-1 proviral integration and gene expression are regulated by the host epigenomic landscape and raise the possibility of reciprocal effects involving host genomic architecture.

### HIV-1 Integration Sites and Host Chromatin Organization

3C-based approaches were used to elucidate viral and host chromatin organization and their relationship to viral gene expression in HIV-1-infected cells. An early 3C study of HIV-1 provirus structure showed loop formation between the 5′ and 3′ long terminal repeats (LTRs), which did not require proviral insertion but was strongly linked to HIV-1 transcription [78]. In addition, 4C-seq data using HIV-1 provirus as the viewpoint demonstrated a frequent chromosomal interaction between integrated HIV-1 and the pericentromeric region of chromosome 12, which correlated with transcriptional silencing in the latent state [79]. Recent Hi-C analysis of HIV-1-infected cells revealed that genes recurrently targeted by the virus are proximal to super-enhancer genomic elements that tend to cluster spatially in the nuclei of CD4^+^ T cells [80]. Because HIV-1 can invade the central nervous system, productively infecting macrophages and microglia therein, the HIV-1 integration pattern in relation to the 3D genomic architecture of microglia cells was also investigated by Hi-C [81]. The results further strengthened the notion that HIV-1 integration preferentially occurs at active, open chromatin, including CTCF-bound regions with active histone marks positioned proximal to TAD boundaries. Finally, a comprehensive analysis of epigenomic, transcriptomic, and 3D genome architecture (Hi-C) datasets in combination with machine learning was undertaken to discern patterns characterizing HIV-1 proviral transcription. Specific chromatin states, active nuclear sub-compartments, and unique positions as well as orientations with respect to human genes and regulatory elements were all found to correlate with proviral transcriptional activity in CD4^+^ T cells [82].

## Influenza Virus

Influenza viruses feature a negative-sense, single-stranded RNA genome and are a common cause of respiratory infections. Transcription and replication of influenza virus occur in the nucleus of host cells and can affect cell function. Interestingly, although the influenza viral genome does not integrate and is not known to impact host chromatin organization via direct interactions, an in situ Hi-C time course of influenza A (IAV)-infected human monocyte-derived macrophages demonstrated that infection significantly alters host 3D genomic architecture [83]. In a subset of highly transcribed host genes, the viral NS1 protein induces read-through transcription by inhibition of transcription termination, which enables RNA polymerase II to disrupt downstream chromatin loops by discharging cohesin from CTCF-bound sites that serve as chromatin anchors, leading to locus decompaction that is more permissive to TF binding (Fig. 2F, left). Another study, using 4C-seq and DNA FISH data, reported an alternative viral strategy targeting cohesin, which is mediated by the IAV protein NP and impacts host chromosomal organization to enhance viral replication [84]. In this scenario, the viral protein NP binds to the host histone H4 methyltransferase Suv4-20h2, disrupting the association of the latter with cohesin. Suv4-20h2 normally suppresses transcription of the *HoxC8-HoxC6* loci by preventing cohesin-dependent looping that has a stimulatory effect on gene expression. Because the encoded HoxC8 and HoxC6 proteins promote IAV replication, active loop formation at the *HoxC8-HoxC6* loci, as a consequence of NP-dependent disruption of the Suv4-20h2-cohesin interaction, contributes to influenza-induced pathology [85] (Fig. 2F, right).

## SARS-CoV-2

Since its emergence in late 2019, SARS-CoV-2, a positive-sense, single-stranded RNA virus, has resulted in >600 million cases of its associated disease COVID-19 and more than ~6.5 million deaths worldwide as of September 2022. Given that the pandemic virus can elicit long-term sequelae in a significant subset of infected individuals, even in the absence of severe disease initially, it is crucial to elucidate the relevant viral-host regulatory strategies in infected cells.

### Host Chromatin Organization and COVID-19 Severity

Although a direct association of the SARS-CoV-2 genome or viral gene products with host chromatin has not been described yet, a few reports suggest a functional interplay between the virus and host chromatin structure. An example of host genomic organization affecting SARS-CoV-2 pathogenesis has been characterized for the *IL-6* locus. The cytokine IL-6 contributes to hyper-inflammation and its levels strongly correlate with the severity of COVID-19. CTCF binding at the *IL-6* enhancer promotes *IL-6* expression. A SNP that disrupts the conserved CTCF-binding site has been shown to attenuate IL-6 induction in COVID-19, thereby providing protection from severe outcomes [86]. Similarly, genome-wide association studies (GWAS) have identified 3p21.31 as the most robust risk locus for severe COVID-19 [87]. At this locus, a collection of chemokine receptors (*CCR1*, *CCR2*, and *CCR5*) and multiple enhancer elements were found to cluster as a CTCF-dependent active chromatin hub [88]. Rapid degradation of CTCF by an auxin-inducible degron (mAID) decommissioned the chromatin hub and selectively reduced expression of the constituent chemokine receptors in macrophages. Because high expression of chemokine receptor *CCR2* in monocytes and macrophage is an established pathologic hallmark of severe COVID-19 [89, 90], CTCF-mediated 3D chromatin interactions play a critical role in the acute respiratory symptoms of COVID-19 and may also directly or indirectly impact other affected tissues.

### Effect of SARS-CoV-2 Infection on Host Chromosome Architecture

Recent reports also suggest that SARS-CoV-2 can affect the host epigenomic landscape and genomic organization. Comprehensive transcriptomic analysis of multiple infected cell lines and COVID-19 patient-derived samples combined with pathway enrichment analysis implicated over 60 epigenetic response proteins that are relevant to SARS-CoV-2 infection [91]. Additionally, differential DNA methylation patterns in immune cells that distinguish COVID-19 convalescents from uninfected controls have been reported [92]. Finally, changes in host 3D chromatin architecture upon SARS-CoV-2 infection have been assessed by in situ Hi-C. Quantification of the pairwise interactions between chromosome regions showed that SARS-CoV-2 infection alters host chromosome organization to induce certain pro-inflammatory genes and suppress antiviral interferon-responsive genes [93]. These changes are accompanied by weakening/switching of A/B compartments and intra-TAD chromatin contacts. In this study, alteration of chromatin compartmentalization was observed in as much as 30% of genomic regions. The molecular basis of this widespread impact of SARS-CoV-2 on host 3D genome architecture may be attributable, at least in part, to selective loss of cohesin at intra-TAD regions, but further investigation will be required to uncover the mechanistic details.

## Conclusion and Outlook

The application of evolving 3C-based technologies combined with high-throughput sequencing in recent years has yielded substantial insights into 3D genomic architectural changes consequent to viral infection, but much remains to be learned. While early findings clearly show the potential for DNA viruses, whether as chromatinized episomes or upon integration, to impact host genomic architecture, RNA viruses, even in the absence of a capacity to integrate, also can elicit alterations in host chromatin structure (Table 2). The molecular mechanisms employed by RNA viruses, which are typically limited to acute infection, are likely varied but may still converge on common host architectural factors, such as CTCF and cohesin. Indeed, recent evidence suggests that IAV and SARS-CoV-2 infections can perturb CTCF and/or cohesin distribution to cause alterations in host chromosomal topology [83, 84, 93], while CTCF/cohesin-dependent host genome organization may contribute to the pathology associated with these RNA viruses [86, 88]. Nevertheless, further investigation is needed to elucidate the mechanistic details by which non-integrating RNA viruses affect host chromatin architecture to modulate gene expression and promote disease. Although RNA viruses generally feature smaller genomes than their DNA counterparts and therefore tend to encode fewer gene products, both viral proteins and non-coding RNAs could contribute to alterations in host chromatin profiles and chromosomal organization. Notably, CTCF localization can be influenced by its RNA-binding capacity, which could have concomitant effects on chromosome conformation [94]. While molecular mechanisms underlying conformational changes in host as well as viral genomes have frequently implicated CTCF and the associated cohesin complex, alternative strategies, involving, for example, condensin, which unlike cohesin does not rely on CTCF to determine its genomic distribution in mammalian genomes [95], YY1 [59] and other factors with possibly unappreciated architectural roles likely await future discovery.

The functional significance of DNA virus-elicited changes in host genomic structure has been linked to oncogenesis and best characterized in cancer cells but almost certainly applies to other pathological states, such as neurological disease. Notably, there is accumulating evidence of a viral etiology for at least a subset of multiple sclerosis (MS) [96] and Alzheimer’s disease (AD) [97, 98] cases, possibly involving specific herpesviruses in each pathological context and likely manifesting in combination with certain genetic susceptibility loci. The potential effects of viral genomes or gene products on host genomic organization, which may be cell-type specific within the brain, and the concomitant altered expression of susceptibility genes have not been addressed in these central nervous system (CNS) pathologies.

The 3C-derived strategies that have informed our current understanding of the impact of viral infection on host 3D genome structure and possible reciprocal effects on viral genomic architecture have clear limitations, primarily related to the necessary inclusion of crosslinking and ligation steps that can introduce artifacts as well as the logistics and cost considerations that allow for sampling of only one or, at best, a few time points. Alternative methods, such as SPRITE and GAM, still require crosslinking and have not been widely adopted yet, possibly due to technical challenges and accessibility. Future insights will likely involve additional approaches, including advanced microscopy methods and analytical pipelines that, when combined with clustered, regularly interspaced, short palindromic repeats (CRISPR)-based genomic modification and visualization strategies [99, 100], can yield simultaneous, real-time data on specific transcriptional events as well as nuclear spatial organization and the structural dynamics of both the host and viral genomes in infected cells.

## Figures and Tables

**Table 1:** Involvement of CTCF/cohesin in viral replication, reactivation, and gene expression.

Virus		Reported viral effect(s) of:	

		CTCF knockdown	Cohesin knockdown
Herpesviruses	HSV1	(HHV1) In lytic infection [40]: ↓ viral gene expression, genome copy number, and viral yield ↓ RNA pol II viral genome occupancy ↑ H3K9me^3^, H3K27me^3^ viral genome deposition In latent infection [101]: ↑ ICP0 expression, ↑ viral reactivation	In lytic infection [41]: ↓ viral gene expression, genome copy number, and viral yield ↓ RNA pol II viral genome occupancy ↑ RNA pol II P-ser5 on viral genes ↑ H3K27me^3^ viral genome deposition
	EBV (HHV4)	No effect on viral reactivation [102]	Loss of long-range interactions within the viral genome in latently infected cells ↑ latent viral gene expression [16]
	KSHV (HHV8)	Modified viral chromatin conformation Altered lytic and latent viral gene expression [103] ↑ viral yield altered viral gene expression [34] lytic gene expression unaffected viral DNA replication unaffected [21]	Modified viral chromatin conformation Altered lytic and latent viral gene expression [103] ↑ viral yield altered viral gene expression [34] ↑ lytic viral gene expression ↑ viral DNA replication [21]
	HCMV (HHV5)	↑ immediate early and early lytic gene expression ↑ viral yield [38]	
HPV		↓ viral replication/amplification [56]	↓ viral replication/amplification [56]
HIV-1		↓ establishment of latency [104]	
HBV		↑ viral gene expression [105]	None with wild-type virus ↑ viral gene expression in the absence of viral transcriptional regulator HBx [106]
Adenovirus		↓ viral replication ↓ late but not early viral gene expression [107]	

**Table 2:** Viral mechanisms involving chromatin architecture.

	Genome	Integration into host	Regulatory mechanisms involving chromatin architecture
HHVs	DNA	No Persists as an episome	● Regulation of viral gene expression via CTCF/cohesin-dependent changes in episomal structure ● Alterations of epigenetic/chromatin organization by viral gene products to modulate host gene expression ● Viral-host chromosomal interactions
HPVs	DNA	Yes Maintained either as an episome or by integration into the host genome	● Regulation of viral gene expression via CTCF/cohesin/YY1-dependent changes in episomal structure ● Activation of viral oncogenes due to disruption of CTCF binding sites during integration ● Hijacking of host enhancers to drive viral oncogene expression ● Alteration of host gene expression by generation of host chromosome SVs upon viral integration
HIVs	RNA	Yes Stable integration into the host genome	● Preferential integration into active open chromatin ● Proviral gene expression is correlated with the epigenomic landscape of the host chromosomal loci that contact the viral integration site
Influenza virus	RNA	No	● CTCF/cohesin-dependent host chromosomal structure affects viral pathogenesis ● Disruption of host genomic organization by viral protein-mediated transcriptional read-through ● Activation of host genes by viral protein-induced cohesin recruitment to the host genomic locus
SARS-CoV-2	RNA	No	● CTCF/cohesin-dependent host chromosomal structure affects viral pathogenesis ● Infection-induced attenuation of chromatin compartmentalization
